# Osilodrostat Treatment of Cushing Syndrome in Real-World Clinical Practice: Findings From the ILLUSTRATE study

**DOI:** 10.1210/jendso/bvaf046

**Published:** 2025-03-15

**Authors:** Maria Fleseriu, Richard J Auchus, Wenyu Huang, Joanna L Spencer-Segal, Kevin C J Yuen, Kelley C Dacus, Julianne Padgett, Elizabeth K Babler, Ashis K Das, Cynthia Campos, Michael S Broder, Adriana G Ioachimescu

**Affiliations:** Departments of Medicine and Neurological Surgery, Pituitary Center, Oregon Health & Science University, Portland, OR 97239, USA; Department of Internal Medicine, Division of Metabolism, Endocrinology and Diabetes, University of Michigan, Ann Arbor, MI 48109, USA; Department of Pharmacology, University of Michigan, Ann Arbor, MI 48109, USA; Division of Endocrinology, Metabolism and Molecular Medicine, Northwestern University Feinberg School of Medicine, Chicago, IL 60611, USA; Department of Internal Medicine, Division of Metabolism, Endocrinology and Diabetes, University of Michigan, Ann Arbor, MI 48109, USA; Michigan Neuroscience Institute, University of Michigan, Ann Arbor, MI 48109-2800, USA; Department of Neuroendocrinology and Neurosurgery, Barrow Pituitary Center, Barrow Neurological Institute, St Joseph's Hospital and Medical Center, Phoenix, AZ 85013, USA; Department of Medicine, University of Arizona College of Medicine and Creighton School of Medicine, Phoenix, AZ 85012, USA; Recordati Rare Diseases Inc, Bridgewater, NJ 08807, USA; Recordati Rare Diseases Inc, Bridgewater, NJ 08807, USA; Recordati Rare Diseases Inc, Bridgewater, NJ 08807, USA; PHAR (Partnership for Health Analytic Research), Beverly Hills, CA 90212, USA; PHAR (Partnership for Health Analytic Research), Beverly Hills, CA 90212, USA; PHAR (Partnership for Health Analytic Research), Beverly Hills, CA 90212, USA; Department of Medicine, Division of Endocrinology and Molecular Medicine, Medical College of Wisconsin, Milwaukee, WI 53226, USA; Department of Neurosurgery, Medical College of Wisconsin, Milwaukee, WI 53226, USA

**Keywords:** Cushing disease, ectopic adrenocorticotropin syndrome, adrenal Cushing syndrome, osilodrostat, retrospective, real world

## Abstract

**Context:**

In clinical trials, osilodrostat (11β-hydroxylase inhibitor) effectively reduced cortisol levels in patients with endogenous Cushing syndrome (CS).

**Objectives:**

A real-world study (ILLUSTRATE) was conducted evaluating osilodrostat use in patients with various etiologies of CS in the United States.

**Methods:**

A retrospective chart-review study was conducted of adults with CS treated with osilodrostat between May 1, 2020, and October 29, 2021.

**Results:**

A total of 42 patients (Cushing disease, n = 34; CS due to adrenal adenoma, n = 5; ectopic adrenocorticotropin syndrome [EAS], n = 3) were included. Starting doses were 2 mg twice daily in 27/42 patients (64.3%), maintenance doses were 2 mg twice daily in 6 of 9 patients (66.7%) attaining them. During osilodrostat treatment, urinary free cortisol (UFC) decreased below the upper limit of normal (ULN) in 14 of 20 patients (70.0%) with pretreatment UFC greater than the ULN. Osilodrostat response was observed across a range of doses (2-20 mg/day). In Cushing disease, median UFC and late-night salivary cortisol decreased from 3.03 and 2.39 × ULN, respectively, to 0.71 and 1.13 × ULN at last assessment in those with available data (n = 17 and 8, respectively). UFC decreased in all patients with adrenal CS or EAS with available data (n = 2 each). There were no unexpected safety signals; the most common adverse events (incidence ≥20%) were fatigue, nausea, and lower-extremity edema. Glucocorticoid withdrawal syndrome and/or adrenal insufficiency were reported in 12 of 42 patients (28.6%) after osilodrostat initiation, resulting in treatment discontinuation in 4.

**Conclusion:**

In routine practice with dosing individualized according to clinical condition, response, and tolerability, osilodrostat was effective and well tolerated regardless of CS etiology and severity.

Endogenous neoplastic Cushing syndrome (CS) is a serious endocrine condition characterized by excessive endogenous cortisol secretion [1, 2]. Untreated, hypercortisolism has serious cardiovascular, metabolic, neuropsychiatric, and infectious consequences, which negatively affect patients' quality of life [2-4]. The risk of mortality is higher in patients with CS than in the general population, mainly because of greater mortality from cardiovascular and infectious diseases [2-5]. More recently, it has been shown that cancer risk is increased in population cohorts with CS [6, 7].

Most cases of CS are caused by excess secretion of adrenocorticotropin (ACTH) from a pituitary adenoma (Cushing disease), which results in excess cortisol release from the adrenal glands [2, 4]. However, some patients may present with ectopic ACTH syndrome (EAS; also referred synonymously as ectopic CS) or ACTH-independent cortisol excess from adrenal adenomas, adrenocortical cancers, or bilateral nodular adrenal hyperplasia (adrenal CS) [2, 4].

Osilodrostat is a potent oral inhibitor of 11β-hydroxylase, the enzyme that catalyzes the final step of cortisol synthesis in the adrenal cortex [8]. In phase 3 trials in patients with Cushing disease, osilodrostat was associated with a rapid and sustained reduction in cortisol levels, as well as improvements in cardiovascular and metabolic parameters, the physical manifestations of Cushing disease, and patients' quality of life [9-13]. The clinical development program for osilodrostat also included a phase 2 study in patients with EAS or adrenal CS, which demonstrated that osilodrostat can lower cortisol regardless of the etiology of CS [14]. Based on these data, osilodrostat was licensed for use in adults with Cushing disease for whom pituitary surgery is not an option or has not been curative (United States) [15] and in adults/patients with endogenous CS (Europe [16]/Japan [17]).

Although prospective clinical trials are essential for demonstrating the efficacy and safety of drug therapies, they entail enrollment of selected patient populations and a tightly controlled research setting [18]. Real-world observational studies, in which the drug is used according to physicians' clinical practice, provide complementary and helpful information in this context. Previous real-world studies conducted in patients with CS in Europe have shown that osilodrostat reduces cortisol levels and improves comorbidities, with no unexpected safety signals [19-22]. The present study, osIlodrostat reaL-worLd Utilization Study To Retrospectively Assess paTient Experience (ILLUSTRATE), was conducted in multiple clinical practices in the United States to evaluate the dosing, effectiveness, and safety of osilodrostat in patients with CS, irrespective of its etiology.

## Materials and Methods

ILLUSTRATE was a retrospective chart-review study of patients in the United States treated with osilodrostat between May 1, 2020, and October 29, 2021. The index date for each patient was defined as the date of the first osilodrostat prescription (between May 1, 2020, and October 29, 2021). Preprescription data were collected from the 12 months before each patient's index date to provide baseline data for that patient.

Patients were eligible for inclusion if aged 18 years or older, with a diagnosis of endogenous neoplastic CS (due to a pituitary adenoma, adrenal adenoma, or ectopic tumor) and a documented prescription for osilodrostat on or after May 1, 2020. As osilodrostat was approved for Cushing disease in the United States earlier the same year (March 6, 2020) [23], the start date for ILLUSTRATE was selected to avoid inclusion of patients treated with osilodrostat in clinical trials. The study was approved by a central independent review board (Western Institutional Review Board). As the study employed secondary data collection of anonymized patient data, a waiver of consent was granted under the privacy rule of HIPPA (the Health Insurance Portability and Accountability Act).

If available, the following data were extracted from patients' medical records into an electronic case report form (eCRF): demographic details (age, sex, race, ethnicity); clinical history (date of CS diagnosis, signs and symptoms, prior surgery and/or radiotherapy); duration of disease prior to osilodrostat prescription; other therapies (cortisol-lowering medications, concomitant steroid use or replacement, antihypertensive and antidiabetic medications); laboratory data (urinary free cortisol [UFC], late-night salivary cortisol [LNSC], morning serum cortisol, serum potassium, testosterone [female patients only]); osilodrostat use (starting dose, uptitration, downtitration, duration of treatment); and adverse events (AEs). AEs were selected from a dropdown list that included the following: hypotension, hyperkalemia, hypokalemia, prolonged QT interval on electrocardiogram, lower-extremity edema, dizziness, rash, constipation, fatigue, alopecia, headache, nausea, vomiting, pituitary tumor size, hypertension, hirsutism, acne, irregular menstruation, brain fog or other cognitive changes, insomnia, striae, muscle weakness, depression, anxiety, other emotional changes, arthralgia/myalgia, and sleep changes.

The following variables were derived from the information recorded in the eCRF: time to maintenance dose (maintenance dose was defined as the first dose that was not modified between two consecutive visits, which could include the baseline visit); titration interval (time between osilodrostat dose changes; if patients had multiple dose changes, the average was reported); and proportion of patients on osilodrostat 6 months after the index prescription. In patients who had dose changes after reaching the maintenance dose, the time between these dose changes was included in the calculation of average titration interval.

Investigator-reported events of glucocorticoid withdrawal syndrome (GWS) and adrenal insufficiency (AI) were evaluated by two of the authors (J.L.S.-S. and K.C.D.) and adjudicated according to symptoms and the level of morning serum cortisol, where available. If the symptoms were consistent with GWS and serum cortisol at the time of the event was greater than 10 µg/dL (>276 nmol/L), these cases were classified as GWS. If the symptoms were more severe or cortisol levels were less than or equal to 10 µg/dL (≤276 nmol/L), AI could not be ruled out, and these cases were classified as such. If the investigator recorded GWS and AI in the same patient at the same visit, they were classified as a single event.

Descriptive statistics were used for all variables based on the number of patients with data available for each variable. Laboratory measures were reported as n times the upper limit of normal (ULN). Independent quality assessments were conducted to check content, inconsistencies, and missing fields in the eCRF.

## Results

Overall, 42 patients were included in the study: 34 patients with Cushing disease, 5 patients with adrenal CS as a result of adrenal adenoma, and 3 patients with EAS. Two patients with Cushing disease had only a single clinical encounter and were included in the baseline results only. Four patients (9.5%), all with Cushing disease, discontinued osilodrostat during follow-up; in all cases, this was because of GWS or AI.

### Baseline Characteristics

Baseline demographics and clinical characteristics are summarized in Table 1. Mean age was 43.7 years, and most patients (76.2%) were female. Mean disease duration before osilodrostat prescription was 57.3 months, and most patients had previously undergone surgery (81.0%) and/or received one or more medical therapies (61.9%). In the subgroup of patients with adrenal CS (n = 5), 2 patients had undergone surgery and 2 patients had expressed a preference not to undergo surgery; in the final patient, information on previous surgery was not recorded in the eCRF. In the overall study population, median UFC, LNSC, and morning serum cortisol levels were 2.54, 2.39, and 1.16 × ULN, respectively. Twelve and 5 patients, respectively, had morning serum cortisol levels and UFC less than the ULN at baseline; of these, 6 (50.0%) and 3 (60.0%), respectively, had received previous medical therapy.

**Table 1.** Baseline demographic and clinical characteristics (overall and by etiology)

	All patients (n = 42)	Cushing disease (n = 34)	Adrenal CS (n = 5)*^a^*	EAS (n = 3)*^b^*
Mean age (SD), y	43.7 (15.0)	40.8 (13.9)	49.2 (14.0)	66.7 (3.5)
Sex, n (%)				
Female	32 (76.2)	27 (79.4)	2 (40.0)	3 (100)
Male	10 (23.8)	7 (20.6)	3 (60.0)	0
Race, n (%)				
White	22 (52.4)	17 (50.0)	4 (80.0)	1 (33.3)
Black or African American	10 (23.8)	8 (23.5)	1 (20.0)	1 (33.3)
Asian	2 (4.8)	1 (2.9)	0	1 (33.3)
Multiracial	1 (2.4)	1 (2.9)	0	0
Unknown	7 (16.7)	7 (20.6)	0	0
Ethnicity, n (%)				
Hispanic, Latino, or Spanish origin				
Yes	8 (19.0)	8 (23.5)	0	0
No	28 (66.7)	20 (58.8)	5 (100)	3 (100)
Unknown	6 (14.3)	6 (17.6)	0	0
Mean age at CS diagnosis (SD), y	37.7 (14.8)	34.9 (12.7)	40.0 (14.8)	66.3 (3.1)
Mean duration of disease prior to osilodrostat prescription (SD), mo	57.3 (82.0)	64.8 (86.7)	30.4 (26.6)	1.2 (0.3)
Previous pituitary or adrenal surgery for CS, n (%)	34 (81.0)	32 (94.1)	2 (40.0)	0
Radiotherapy for CS in last 5 y, n (%)	10 (23.8)	10 (29.4)	0	0
Previous medical therapy for CS,*^c^* n (%)	26 (61.9)	21 (61.8)	3 (60.0)	2 (66.7)
Pasireotide	3 (7.1)	3 (8.8)	0	0
Cabergoline	7 (16.7)	7 (20.6)	0	0
Ketoconazole	10 (23.8)	8 (23.5)	0	2 (66.7)
Metyrapone	2 (4.8)	1 (2.9)	1 (20.0)	0
Mitotane	1 (2.4)	0	1 (20.0)	0
Mifepristone	6 (14.3)	5 (14.7)	1 (20.0)	0
UFC,*^d^* × ULN	n = 32	n = 25	n = 4	n = 3
Mean (SD)	7.67 (14.84)	3.14 (2.98)	13.93 (15.25)	37.03 (36.46)
Median (min-max)	2.54 (0.09-75.20)	2.28 (0.09-11.17)	13.77 (0.42-27.76)	33.33 (2.57-75.20)
LNSC,*^d^* × ULN	n = 18	n = 16	n = 1	n = 1
Mean (SD)	4.80 (8.18)	5.25 (8.59)	0.55	1.82
Median (min-max)	2.39 (0.44-36.33)	2.78 (0.44-36.33)	0.55	1.82
Morning serum cortisol,*^d^* × ULN	n = 32	n = 24	n = 5	n = 3
Mean (SD)	1.23 (0.77)	1.13 (0.42)	1.08 (0.97)	2.33 (1.78)
Median (min-max)	1.16 (0.19-4.38)	1.18 (0.19-1.88)	0.83 (0.39-2.76)	1.43 (1.17-4.38)
Potassium levels,*^d^* mmol/L	n = 41	n = 33	n = 5	n = 3
Mean (SD)	4.1 (0.6)	4.3 (0.5)	3.7 (0.5)	3.6 (0.9)
Median (min-max)	4.0 (2.6-5.6)	4.2 (3.6-5.6)	3.4 (3.3-4.5)	3.7 (2.6-4.4)
Potassium levels <LLN,*^e^* n (%)	n = 37 5 (13.5)	n = 30 1 (3.3)	n = 5 3 (60.0)	n = 2 1 (50.0)
Testosterone levels,*^f^* × ULN	n = 11	n = 9	n = 1	n = 1
Mean (SD)	1.00 (1.48)	0.96 (1.56)	0.07	2.29
Median (min-max)	0.36 (0.03-5.02)	0.36 (0.03-5.02)	0.07	2.29

ULNs varied between study centers, ranging from 32 to 64 µg/24 hours (88.3-176.6 nmol/L) for UFC, 0.01 to 0.112 µg/dL (0.28-3.09 nmol/L) for LNSC, 18.4 to 25 µg/dL (507.8-690.0 nmol/L) for morning serum cortisol, and 41 to 100 ng/dL (1.42-3.47 nmol/L) for testosterone (female patients).

Abbreviations: CS, Cushing syndrome; EAS, ectopic adrenocorticotropin syndrome; LLN, lower limit of normal; max, maximum; min, minimum; ULN, upper limit of normal.

^a^All patients had adrenal adenoma.

^b^In 1 patient, the primary tumor could not be located; in the other 2 patients, the location of the ectopic tumor was not recorded.

^c^Reasons for stopping these therapies and switching to osilodrostat were not collected as part of this study.

^d^Not all patients had values recorded at baseline (see n numbers in each column).

^e^LLN was not available for all patients with available potassium data.

^f^Female patients only.

### Osilodrostat Dosing

**Figure 1.**
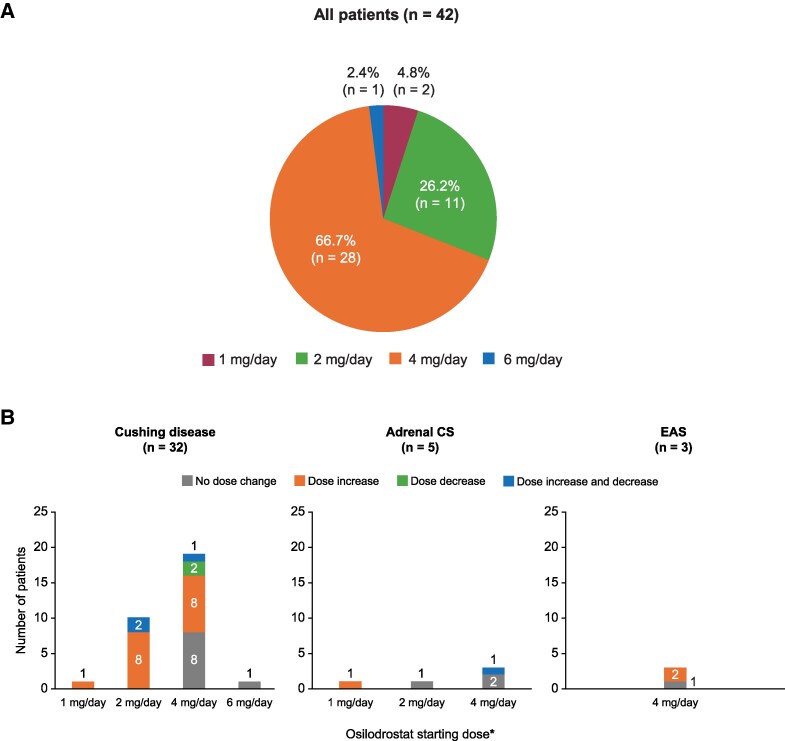
A, Total osilodrostat daily starting dose* in all patients (n = 42) and B, dose changes postinitiation (by etiology) in patients with at least one clinical encounter after initiating osilodrostat (n = 40). *Osilodrostat starting doses were given twice daily in all except 3 patients with Cushing disease (initiated on 1 mg once daily, 2 mg once daily, and 4 mg once daily) and 1 patient with adrenal CS (initiated on 1 mg once daily).

Abbreviations: CS, Cushing syndrome; EAS, ectopic adrenocorticotropin syndrome.

Information on osilodrostat dosing is summarized in Table 2. The most common starting dose of osilodrostat was 4 mg/day in 28 of 47 patients (66.7%; Fig. 1A), comprising 2 mg twice daily in 27 patients and 4 mg once daily in 1 patient. Some patients with Cushing disease and adrenal CS were initiated on lower doses (1 mg once daily [n = 2] or twice daily [n = 10], or 2 mg once daily [n = 1]).

**Table 2.** Osilodrostat treatment (overall and by etiology)

	All patients (n = 42)	Cushing disease (n = 34)	Adrenal CS (n = 5)	EAS (n = 3)
No. of patients with initial dose information	42	34	5	3
Starting total daily dose, mg				
Mean (SD)	3.4 (1.1)	3.4 (1.1)	3.0 (1.4)	4.0 (0.0)
Median (min-max)	4 (1-6)	4 (1-6)	4 (1-4)	4 (4-4)
Starting dose schedule, n (%)				
Twice daily	38 (90.5)	31 (91.2)	4 (80.0)	3 (100)
Once daily	4 (9.5)	3 (8.8)	1 (20.0)	0
Starting dose and schedule, n (%)				
1 mg once daily	2 (4.8)	1 (2.9)	1 (20.0)	0
1 mg twice daily	10 (23.8)	9 (26.5)	1 (20.0)	0
2 mg once daily	1 (2.4)	1 (2.9)	0	0
2 mg twice daily	27 (64.3)	21 (61.8)	3 (60.0)	3 (100)
3 mg twice daily	1 (2.4)	1 (2.9)	0	0
4 mg once daily	1 (2.4)	1 (2.9)	0	0
No. of patients with postinitiation clinical encounters	40	32	5	3
Patients who reached maintenance dose by last interaction, n (%)	9 (22.5)	7 (21.9)	1 (20.0)	1 (33.3)
Time to maintenance dose or end of follow-up, wk				
Mean (SD)	34.7 (18.0)	36.7 (19.1)	23.0 (11.7)	33.6 (3.3)
Median (min-max)	33 (0.7-78.1)	37 (0.7-78.1)	17 (13.0-37.6)	32 (31.3-37.4)
Maintenance dose,*^a^* n (%)				
2 mg twice daily	6 (66.7)	4 (57.1)	1 (100)	1 (100)
4 mg twice daily	2 (22.2)	2 (28.6)	0	0
10 mg twice daily	1 (11.1)	1 (14.3)	0	0
Patients with dose change, n (%)	25 (62.5)	21 (65.6)	2 (40.0)	2 (66.7)
Titration period in patients with dose change, wk				
Mean (SD)	13.8 (12.4)	14.8 (13.2)	4.9 (3.2)	13.0 (0.0)
Median (min-max)	11 (2.6-57.0)	11 (4.6-57.0)	5 (2.6-7.1)	13 (13.0-13.0)
Osilodrostat treatment interruption,*^b^* n (%)	9 (22.5)	7 (21.9)	1 (20.0)	1 (33.3)
Duration of exposure up to treatment interruption or study end, wk				
Mean (SD)	35.2 (22.0)	36.5 (22.5)	26.8 (26.3)	35.8 (4.5)
Median (min-max)	35 (0.7-78.1)	38 (0.7-78.1)	14 (4.0-69.7)	37 (30.7-39.3)
Duration of treatment up to study end,*^c^* wk				
Mean (SD)	37.0 (20.9)	38.2 (21.5)	29.4 (24.0)	36.0 (4.2)
Median (min-max)	37 (0.7-78.1)	40 (0.7-78.1)	17 (13.0-69.7)	37 (31.3-39.3)
Patients on osilodrostat for ≥6 mo prior to study end,*^c,d^* n (%)	28 (96.6)	23 (95.8)	2 (100)	3 (100)
Duration of therapy prior to study end*^c^* in patients with ≥6 mo persistence, wk				
Mean (SD)	47.3 (15.2)	48.5 (15.3)	51.6 (25.6)	36.0 (4.2)
Median (min-max)	44.9 (28.1-78.1)	45.7 (28.1-78.1)	51.7 (33.6-69.7)	37.4 (31.3-39.3)

Abbreviations: CS, Cushing syndrome; EAS, ectopic adrenocorticotropin syndrome.

^a^In those who reached a maintenance dose (defined as a dose that was not modified between 2 consecutive visits) by last interaction.

^b^Defined as a break in osilodrostat treatment for 30 days or longer.

^c^Or treatment discontinuation (n = 4).

^d^In 29 patients with 6 months' follow-up (n = 24, 2, and 3 for Cushing disease, adrenal CS, and EAS, respectively).

Mean titration interval was 13.8 weeks for all patients (4.9 weeks in patients with adrenal CS, 13.0 weeks in patients with EAS, and 14.8 weeks in patients with Cushing disease). Nine patients (22.5%; 7 patients with Cushing disease and 1 patient each with adrenal CS and EAS) achieved a maintenance dose of osilodrostat (defined as no dose modification between 2 consecutive visits) after a mean (SD) of 34.7 (18.0) weeks. The most common maintenance dose was 2 mg twice daily (see Table 2).

Overall, 25 of 40 patients (62.5%) had their osilodrostat dose adjusted during the study. In most of these cases (n = 20), the dose was increased; 2 patients had a dose decrease and 3 patients had doses both increased and decreased (Fig. 1B). In those who had an osilodrostat dose increase only, most (14/20; 70.0%) had only a single dose increase; in the remaining patients, 4 (20.0%) had 2 dose increases and 2 (10.0%) had 4 dose increases. In those who had dose adjustments, there was no observable pattern between starting and final osilodrostat doses (Table 3). Overall, 9 of 40 patients (22.5%) had osilodrostat treatment temporarily interrupted, which occurred in the first 3 months of treatment in 5 patients, between treatment months 3 and 6 in 2 patients, and after 6 months in 2 patients. Reasons for temporary interruption were hypocortisolism-related AEs, patient undergoing surgery, lack of availability of osilodrostat in the hospital setting, and insurance noncoverage.

**Table 3.** Shift table showing starting and final doses for those patients who had dose adjustments during the study period (n = 26)

Starting dose*^a^*	Final dose, No. of patients								
	1 mg/d	2 mg/d	4 mg/d	5 mg/d	6 mg/d	8 mg/d	10 mg/d	14 mg/d	20 mg/d
**Cushing disease** *^b^*									
1 mg/d (n = 1)		1							
2 mg/d (n = 10)			8		1		1		
4 mg/d (n = 11)	1	1	1			4		2	2
**Adrenal CS**									
1 mg/d (n = 1)			1						
4 mg/d (n = 1)			1						
**EAS**									
4 mg/d (n = 2)				1		1			

Some patients had the same starting and final doses as their doses were increased then decreased during the study.

Abbreviations: CS, Cushing syndrome; EAS, ectopic adrenocorticotropin syndrome.

^a^Osilodrostat starting doses were given twice daily in all except 3 patients with Cushing disease (initiated on 1 mg once daily, 2 mg once daily, and 4 mg once daily) and 1 patient with adrenal CS (initiated on 1 mg once daily).

^b^One patient had only their osilodrostat starting dose reported.

Mean (SD) duration of follow-up was 37.1 (20.5) weeks, and mean (SD) duration of osilodrostat treatment was 37.0 (20.9) weeks; almost all patients with postinitiation clinical encounters (96.6%) received treatment for 6 months or longer (see Table 2).

### Changes in Cortisol Levels During Osilodrostat Treatment

**Figure 2.**
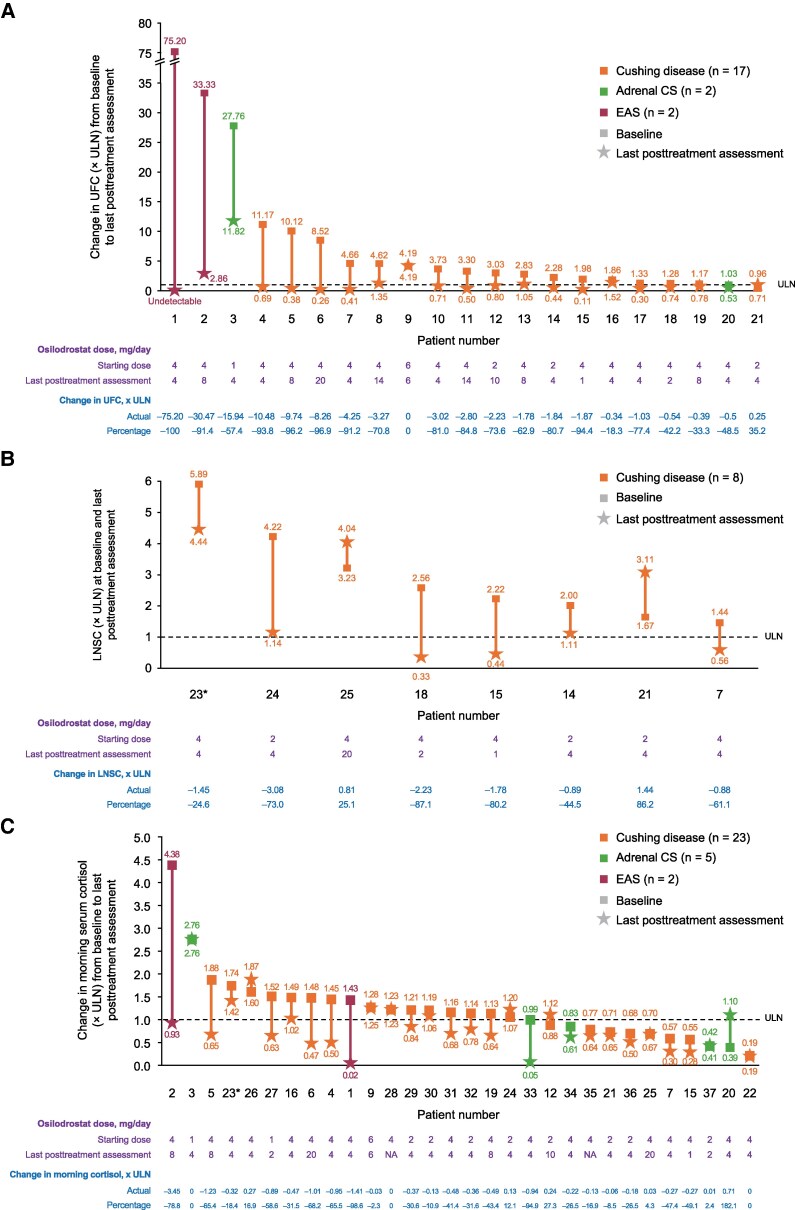
Changes in A, UFC; B, LNSC; and C, morning serum cortisol in individual patients. *In patient 23, the first and last doses recorded were both 2 mg twice daily; during the follow-up period, 2 changes in dose were recorded the same day on 2 separate occasions.

Abbreviations: CS, Cushing syndrome; EAS, ectopic adrenocorticotropin syndrome; LNSC, late-night salivary cortisol; NA, not available; UFC, urinary free cortisol; ULN, upper limit of normal.

In patients with available assessments, median values for all cortisol parameters decreased during osilodrostat treatment, regardless of CS etiology (Table 4). In the subgroup of patients with Cushing disease, median UFC and morning serum cortisol levels were less than the ULN at the last assessment (0.71 and 0.68 × ULN, respectively), while median LNSC levels were slightly higher than the ULN (1.13 × ULN). In patients with UFC, LNSC, and morning serum cortisol levels greater than the ULN at baseline, 12 of 16, 3 of 8, and 8 of 15, respectively, had levels less than the ULN during osilodrostat treatment (Fig. 2); the final osilodrostat doses in these patients ranged from 1 to 20 mg/day. Neither of the patients who had a final dose of 20 mg/day were receiving concomitant glucocorticoids (ie, they were not being treated with a “block-and-replace” approach). In those with UFC and morning serum cortisol less than the ULN at baseline, levels remained less than the ULN in 1 of 1 and 7 of 8 patients, respectively.

**Table 4.** Median (minimum-maximum) urinary free cortisol, late-night salivary cortisol, and morning serum cortisol levels (overall and by etiology)

	All patients	Cushing disease	Adrenal CS	EAS
UFC, × ULN	n = 21	n = 17	n = 2	n = 2
Baseline	3.73 (0.09-75.20)	3.03 (0.09-11.17)	14.40 (1.03-27.76)	54.27 (33.33-75.20)
Last assessment	0.71 (0.02-11.82)	0.71 (0.07-4.19)	6.18 (0.53-11.82)	1.44 (0.02-2.86)
LNSC, × ULN	n = 8	n = 8	n = 0	n = 0
Baseline	2.39 (1.44-5.89)	2.39 (1.44-5.89)		
Last assessment	1.13 (0.44-4.44)	1.13 (0.44-4.44)		
Morning serum cortisol, × ULN	n = 30	n = 23	n = 5	n = 2
Baseline	1.15 (0.19-4.38)	1.16 (0.19-1.88)	0.83 (0.39-2.76)	2.91 (1.43-4.38)
Last assessment	0.67 (0.02-2.76)	0.68 (0.19-1.87)	0.61 (0.05-2.76)	0.47 (0.02-0.93)

Results are based on patients with both baseline and postosilodrostat prescription data available.

Abbreviations: CS, Cushing syndrome; EAS, ectopic adrenocorticotropin syndrome; LNSC, late-night salivary cortisol; UFC, urinary free cortisol; ULN, upper limit of normal.

The effect of osilodrostat on UFC and morning serum cortisol levels in individual patients with adrenal CS or EAS and available data are shown in Fig. 2A and 2C, respectively. In patients with adrenal CS, UFC levels were reduced in the 2 patients with data available, and morning serum cortisol levels were reduced in 2 of 5 patients with data available. In patients with EAS, there were substantial reductions both in UFC and morning serum cortisol (n = 2).

**Figure 3.**
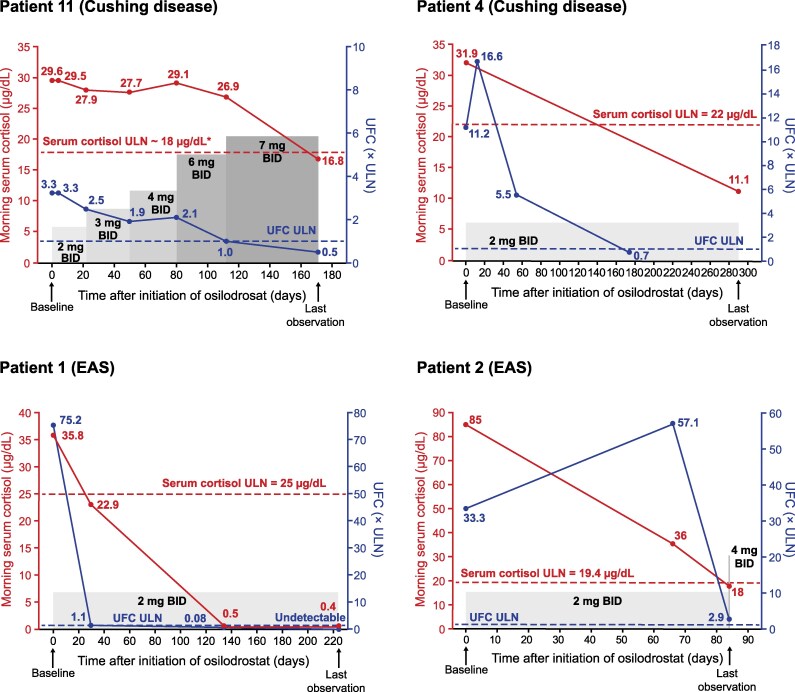
Individual osilodrostat dosing, UFC, and serum cortisol levels during the study period in illustrative patients. ULN for UFC was 50 μg/24 hours for patients 11, 4, and 1 and 42 μg/24 hours for patient 2. *ULN for serum cortisol was not provided for this patient, so it was estimated to be approximately 18 µg/dL.

Abbreviations: BID, twice daily; EAS, ectopic adrenocorticotropin syndrome; UFC, urinary free cortisol; ULN, upper limit of normal.

The time course of cortisol changes and corresponding osilodrostat doses in 2 patients with Cushing disease and 2 patients with EAS are illustrated in Fig. 3.

### Safety and Tolerability

Overall, 29 patients (69.0%) had an AE reported. The most common AEs (incidence ≥20%) were fatigue, nausea, and lower-extremity edema (Table 5).

**Table 5.** Adverse events reported during osilodrostat treatment (overall patient population)*^a^*

AE, n (%)	n = 42*^a^*
Any AE	29 (69.0)
Fatigue	23 (54.8)
Nausea	12 (28.6)
Lower-extremity edema	11 (26.2)
Headache	6 (14.3)
Dizziness	6 (14.3)
Hypokalemia	6 (14.3)
Alopecia	4 (9.5)
Vomiting	3 (7.1)
Hypotension	2 (4.8)
Hyperkalemia	1 (2.4)
Prolonged QT interval on electrocardiogram	1 (2.4)

Abbreviation: AE, adverse event.

^a^Includes 2 patients who had no postinitiation interaction before study end.

According to the events reported by the investigators, 13 patients had GWS (n = 3), AI (n = 3), or both (n = 7). Of these, osilodrostat treatment was interrupted in 3 patients, the dose was decreased in 2 patients, and there was no change in dose in 4 patients; in the remaining 4 patients, osilodrostat treatment was discontinued. Glucocorticoid use was reported in 4 patients overall; this included 2 patients in whom AI and/or GWS were reported. The first patient reported to have AI and GWS by the investigator was prescribed hydrocortisone 5 mg twice daily for 2 weeks. The second patient (AI) was also prescribed hydrocortisone at a dose of 20 mg/day (duration not specified).

On author adjudication, using the criteria outlined in “Materials and Methods,” 1 case of AI was reclassified as GWS and 5 cases reported to be both GWS and AI by the investigator were reclassified as AI only. Another case of AI was excluded as the symptoms of AI had started before initiation of osilodrostat; this patient had been treated with pasireotide in the 5 months before starting osilodrostat. Thus, the number of author-adjudicated cases during osilodrostat treatment was 12: 4 for GWS, 6 for AI, and 2 for both (ie, patients experiencing ≥1 distinct episode of GWS and AI).

To illustrate different presentations and management practices of AI and GWS, we describe details regarding individual cases. In 1 patient with GWS, symptoms were dizziness, nausea, and fatigue, with serum cortisol levels remaining above 10 µg/dL (276 nmol/L). The symptoms occurred early in the course of treatment and were managed by reducing the osilodrostat dose. In another patient with GWS, symptoms occurred after prolonged treatment, and osilodrostat was discontinued. In 2 patients with AI, symptoms were also dizziness, nausea, and fatigue, but serum cortisol levels were less than or equal to 10 µg/dL (≤276 nmol/L). In the first patient, osilodrostat was maintained at the same dose; symptoms resolved and cortisol levels increased slightly, to above 10 µg/dL (276 nmol/L). Treatment was interrupted in the second patient, but further information on the symptoms and cortisol levels were unavailable.

In patients with available data at baseline and last assessment (n = 38), median (minimum-maximum) serum potassium levels were 4.0 (2.6-5.6) and 4.3 (3.7-5.5) mmol/L, respectively. In those who were normokalemic at baseline (n = 30), 2 patients had potassium levels less than the lower limit of normal during osilodrostat treatment; in those who were hypokalemic at baseline (n = 4), 3 patients had potassium levels reverting to normal during treatment. In female patients with available data at baseline and last assessment (n = 4), mean testosterone levels were 1.48 × ULN at baseline and 1.52 × ULN at the last posttreatment assessment.

## Discussion

This study evaluated the real-world use of osilodrostat in patients in the United States with CS during the period shortly after osilodrostat was approved by the US Food and Drug Administration for the treatment of adults with Cushing disease for whom pituitary surgery is not an option or has not been curative. The results highlight the importance of selecting a starting dose and titration regimen according to each patient's circumstances, clinical response, and tolerability, as the dose required to normalize cortisol levels varies between patients and does not appear to depend on baseline levels. Most of the patients in the study (n = 34/42 [81.0%]) had Cushing disease, as expected. In this subgroup, most patients (n = 21/34 [61.8%]) were initiated on the approved starting dose of osilodrostat (2 mg twice daily), but many (n = 11/34 [32.4%]) were started on lower doses. All patients with Cushing disease who were initiated on doses lower than 2 mg twice daily required a dose increase during the study. A dose of 2 mg twice daily was also the most common maintenance dose in those with Cushing disease who reached a maintenance dose: a total of 57.1% remained on 2 mg twice daily, while 28.6% and 14.3% were uptitrated to 4 mg twice daily and 10 mg twice daily, respectively. In phase 3 clinical trials of osilodrostat in patients with Cushing disease (LINC 3 and LINC 4), median (interquartile range) average doses at the end of the extension periods were 7.4 (3.5-13.6) mg/day in LINC 3 and 4.6 (3.7-9.2) mg/day in LINC 4 [9, 11]. In the present study, most patients whose dose was uptitrated required only one dose increase. Mean duration of the titration period in patients with Cushing disease, defined as the period between any osilodrostat dose changes, was 14.8 weeks. In LINC 3 and LINC 4, doses were increased according to UFC levels in the first 12 weeks, after which dose adjustments were permitted during the remainder of the 48-week core period based on efficacy and tolerability [10, 12]. In both trials, starting and final osilodrostat doses were lower in Asian patients with Cushing disease than in non-Asian patients, regardless of body mass index, likely because of differences in bioavailability [24].

In the subgroup of patients with Cushing disease, most of those with UFC and morning serum cortisol levels greater than the ULN at baseline had their levels reduced to normal during treatment (12/16 and 8/15, respectively). Some patients had UFC and morning serum cortisol levels less than the ULN when osilodrostat was initiated, presumably reflecting a switch to osilodrostat from another medical therapy (eg, because of tolerability issues). More than 60% of patients had received one or more medical therapies before starting osilodrostat, but the reasons for stopping these therapies were not collected as part of the study. In patients with normal levels at study baseline, levels remained less than the ULN in 1 of 1 patient for UFC and 6 of 8 patients for morning serum cortisol. Median levels of UFC and morning serum cortisol in the subgroup of patients with Cushing disease decreased to within the normal range during osilodrostat treatment. These real-world results are consistent with those from the 48-week core phases of LINC 3 and LINC 4 [10, 12, 13].

The present study also included a small number of patients with adrenal CS (n = 5) and EAS (n = 3). The starting dose was 2 mg twice daily in all patients with EAS; in patients with adrenal CS, the starting dose was 2 mg twice daily (n = 3), 1 mg twice daily (n = 1), and 1 mg once daily (n = 1). Baseline median UFC, which was higher in the adrenal CS and EAS subgroups than in patients with Cushing disease, decreased during osilodrostat treatment but remained above the ULN. Median morning serum cortisol levels decreased from above to within the normal range in patients with EAS and remained within the normal range in patients with adrenal CS. Notably, baseline levels of morning serum cortisol, mean UFC, or LNSC did not necessarily predict the osilodrostat dose needed for biochemical normalization. Again, these results emphasize the need to individualize the starting dose and titration regimen based on each patient's clinical circumstances and response to treatment. Differences in the pathophysiology of CS subtypes may also influence decisions about osilodrostat titration; for example, unlike in Cushing disease, ACTH rarely rises during inhibition of cortisol synthesis in EAS, and the rise in ACTH is delayed after cortisol normalization in adrenal CS. The results for the adrenal CS and EAS subgroups in the present study should be interpreted with caution given the small number of patients, and data from individual patients are perhaps more illustrative in this context. Individual UFC and morning serum cortisol levels were reduced in all patients with adrenal CS or EAS except for 2 patients (both with adrenal CS), whose morning serum cortisol levels did not change; in one of these patients, morning serum cortisol was within the normal range at baseline. Data on LNSC levels should also be interpreted with caution given the small number of patients with data available for this parameter.

The safety profile of osilodrostat was similar to that observed in clinical trials [10, 12, 14], with no unexpected safety signals. GWS and AI are recognized as potential side effects of osilodrostat based on its mechanism of action, but they can be difficult to differentiate as many of their symptoms overlap [25]. In the present study, there were 12 cases of GWS and/or AI after initiation of osilodrostat (28.6% of patients); this rate is lower than in the LINC 3 clinical trial (54.0% of patients with hypocortisolism-related AEs) [9] and similar to that seen in LINC 4 (27.4%) [11]. In LINC 3 and LINC 4, most hypocortisolism-related AEs were classified by the investigator as AI [11], but further evaluation to differentiate between this and GWS was not possible. For the present study, we were able to evaluate the symptoms and biochemical changes associated with these AEs, and we confirmed that it was often not possible for clinicians to accurately distinguish GWS and AI based on symptoms alone. As GWS and AI may require different management approaches, measurement of serum cortisol levels is essential to help guide the management strategy in these cases. In the present study, most cases of GWS and AI were managed by reducing the dose or temporarily interrupting treatment, which is consistent with the results from LINC 3 and LINC 4 [9-12]; only 4 patients (9.5%) with AI or GWS in the present study had discontinued treatment as a result. Treatment with glucocorticoids was recorded in 2 patients; the timing of treatment indicates that glucocorticoids were used to treat the symptoms of AI/GWS rather than as part of a “block-and-replace” strategy, in which glucocorticoids are administered concomitantly with osilodrostat [21, 26]. Slower dose escalation may reduce the risk of hypocortisolism-related AEs; the longer titration interval in LINC 4 (every 3 weeks) than in LINC 3 (every 2 weeks) may explain the lower incidence of hypocortisolism-related AEs during LINC 4 [10, 12]. Regardless, and as with all steroidogenesis inhibitors, all patients treated with osilodrostat should be monitored regularly and educated on the signs and symptoms of GWS and AI. Case reports have described rare episodes of delayed cortisol reduction during chronic osilodrostat therapy and prolonged AI after its discontinuation, which emphasizes the importance of lifelong, close monitoring of symptoms and serum cortisol levels [27-29].

The results of the present study are consistent with those of previous studies demonstrating the efficacy and safety of osilodrostat in patients with EAS or adrenal CS. In a prospective phase 2 study conducted in Japan, mean UFC levels decreased in all patients and normalized in most following 12 weeks of treatment [14]. In LINC 7, a retrospective study conducted in France, 103 patients with adrenal CS or EAS were followed up retrospectively for up to 36 months; at last assessment, mean UFC was normalized in most patients [30]. Compared with LINC 7, the starting and maintenance doses of osilodrostat were lower in the present study, once again emphasizing the importance of individualizing osilodrostat dosing. In a second real-world study in France, which was conducted in 30 patients with EAS, median UFC decreased significantly following osilodrostat monotherapy (when used both first and second line) and combination therapy with other cortisol-lowering drugs [21]. There were also substantial improvements in hypertension, hyperglycemia, and hypokalemia, allowing the discontinuation or dose reduction of concomitant treatments. Several other case series and case reports have documented effective control of cortisol levels and good tolerability in patients with adrenal CS or EAS [26, 31-38], adding to the body of evidence supporting the use of osilodrostat in patients with CS irrespective of severity and etiology.

Limitations of the present study include the small patient numbers, particularly in the groups with adrenal CS and EAS. As the study was conducted shortly after osilodrostat was approved in the United States for patients with Cushing disease for whom pituitary surgery is not an option or has not been curative, the predominance of patients with Cushing disease was expected, albeit slightly higher (81%) than the overall prevalence of Cushing disease (up to 70%), vs other causes of CS in a clinical setting [2]. Another limitation is that data were extracted retrospectively from patients' medical records rather than recorded prospectively according to a study protocol; this resulted in many missing data points, especially for laboratory data. Despite these limitations, the results provide invaluable information on the real-world use of osilodrostat in the United States, for which data are currently lacking. The design of the study also allowed adjudication of AI vs GWS events by the authors in some patients as morning cortisol levels were available. In addition, ILLUSTRATE is the only completed multicenter study in the United States to date that evaluates the effect of osilodrostat not only in Cushing disease, but also in adrenal CS and EAS, providing data on the cortisol-lowering effectiveness of osilodrostat across the spectrum of hypercortisolism etiologies.

### Conclusions

Results of this real-world study are consistent with those from clinical trials and other real-world studies, showing that osilodrostat was effective and well tolerated in patients with varying etiologies and severities of CS when used by physicians in routine clinical practice. The results also highlight the importance of individualizing the osilodrostat dose and titration regimen according to each patient's clinical condition, response, and tolerability.

## Data Availability

Some data sets generated and/or analyzed during the present study are not publicly available but are available from the corresponding author on reasonable request.

## Abbreviations

ACTH: adrenocorticotropin
AE: adverse event
AI: adrenal insufficiency
CS: Cushing syndrome
EAS: ectopic adrenocorticotropin syndrome
eCRF: electronic case report form
GWS: glucocorticoid withdrawal syndrome
ILLUSTRATE: osIlodrostat reaL-worLd Utilization Study To Retrospectively Assess paTient Experience
LLN: lower limit of normal
LNSC: late-night salivary cortisol
UFC: urinary free cortisol
ULN: upper limit of normal
